# Does the Present Method of Teaching by Didactic Lectures Best Qualify the Student for the Practice of His Profession?

**Published:** 1898-01

**Authors:** John S. Marshall

**Affiliations:** Chicago


### Does the Present Method of Teaching by Didactic Lectures Best Qualify the Student for the Practice of His Profession?

BY DR. JOHN S. MARSHALL, CHICAGO.

Abstract of paper read before Southern Dental Association, Old Point Comfort, August, 1897.

Dr. Marshall answers the question embodied in the title of his paper in the negative, for the following reasons:

First—There are very few men in the profession who have the eminent qualifications to make successful teachers under this system. The paramount qualifications for the office of teacher are—the ability to impart knowledge, and the ability to teach the student how to apply it; and there are very few who possess both of these qualifications.

Second—The majority of the teachers in the dental schools are busy practitioners, who have neither the time nor the energy to properly prepare for the duties of the lecture-room.

Third—The average student can only remember a few of the most striking thoughts presented in each lecture.

The fallibility of the lecture system is seen in the fact that, as a rule, the students show the greatest weakness at the time of examination in those subjects in which they have had to depend upon a course of lectures for information. Listening to a course of lectures and studying the same subject from a suitable text-book are not to be compared as a means of education.

To remedy these defects Dr. Marshall suggests that a full and complete syllabus of each lecture should be furnished the student, by the aid of which he can follow the lecture during its delivery; the syllabus also to be used instead of a quiz compendium. By these means the work in the lecture-room and in the quiz-room would harmonize, and the utmost good possible be got out of this system. The recitation system, however, would be far more satisfactory upon all subjects that are not purely clinical or manipulative. By the use of text-books a systematic plan of study is followed, and the subject-matter is properly classified. By the adoption of this method the younger men could be placed in charge of the instructions in the recitation-room, with orders to follow the authorized text-book, while the application of the knowledge thus gained—the most important part of the work would be in the hands of the professors in the laboratories and clinic-rooms.

Dr. Marshall concluded his paper with the questions:—Are these suggestions too radical? Are they based upon common sense? Are they practical?

ABSTRACT OF DISCUSSION.

In the discussion of Dr. Marshall's paper, Dr. Taft took the position that as improved methods of instruction are being introduced all the way from the kindergarten to the university, there was no reason why the methods of the dental colleges should not be changed, if, thereby, they could be made more efficient. Those who can look back forty or fifty years will recognize that great advances have been made upon the primitive, elementary methods of those days, great changes wrought. In entering upon any line of life certain qualifications are demanded. This rule has not been enforced in our profession. The demand has been for the greatest number in the classes, regardless of fitness or preliminary training. More discrimination is now being exercised. It has come to be recognized that to make available any line of special education, the general education must be as thorough as possible. The people are waking up to the realization of the necessity of preliminary education as essential to success in any line of life. In the dental profession, as much as in law or medicine, progress is in proportion to preliminary preparation. In dentistry, it is not only finger-craft that is needed, but there must be inculcation of principles as well. The lecture method should not be abandoned altogether. With too much study of text-books the mind gets into grooves; follows in the line some one else has worked out; there is no incentive to original thought. The object of education is to stimulate thought, to draw out; not to do just as some one else has done. It is a mistake to discredit the lecture system. The voice and the personality of the lecturer contribute elements not to be found in text-books. Text-books arouse no enthusiasm; they stamp no living impress upon the mind or the character of the student.

DR. M. C. KELLOPS: The object of education is to enable a man to put in practice what he has learned. A common-school education holds good the world over. But in the matter of dental education each State in this country is fighting every other State. I want the right to practice, as I have the right to live—wherever I please.

DR. W. C. BARRETT: There is a mistake in the conception of the meaning of the word "educate." It is not to draw out; how can we draw out what has never been put in? The teacher must have the power of impressing upon others his individuality, his energy, his ambition. He must be a man of imperative strength of character. To be qualified for his work he must be thoroughly familiar with his subject. By the magnetism of his own presence he impresses the students as no book, no pale, cold page, nor task to be learned can ever do. Books are dry; lessons are a task, but the principles inculcated and stamped in by an enthusiastic teacher will make an undying impress upon the mind and character of the student. The mere pedagogue teaches only; he ceases to advance. The didactic system is best for us at the present time when we are teaching what is changing from week to week, from day to day, almost from hour to hour; it is not yet crystalized into text-books.

DR. J. Y. CRAWFORD: A man to be a good dental surgeon needs to be better educated than for any other profession, and he ought to remain longer in college, because his future occupation offers a barrier to subsequent intellectual development. He is going to have the hardest work to make a living. So much of his time will have to be devoted to manual labor that he will have little opportunity to exercise his mental faculties. Therefore, a man should be an educated man before he becomes a dentist. A man who is fitted for professional life differs from the common herd; he stands upon a higher plane. We must quit taking in men out of the workshops; it won't do to try and make doctors of divinity out of everybody. A tower which is top-heavy will fall over into the ditch.

Dr. Marshall, in closing the discussion, said that while he agreed with Dr. Taft that the didactic method was best in certain lines, he thought the fundamental principles of anatomy, physiology, etc., were best taught by the recitation system. Another aspect of the question is that the average college professor of to-day is also a busy practitioner. He has not the time to prepare a series of lectures as they should be prepared. Therefore, let him avail himself of text-books, the best that are to be had of course. Our teachers will never be able to do the best that should be done until some rich men endow our colleges, and so enable such men as Dr. Black, and a few others that could be named, to devote all their time to scientific investigation. There are text-books in anatomy, physiology, etc., that are far from being primers or kindergarten work; books that would demand serious, earnest study. Teachers should also do more in the line of teaching students how to apply what they have learned.

